# Modification of Wheat Bran by Solid-State Fermentation with *Wolfiporia cocos*: Enhanced Bioactive Release, Reduced Anti-Nutritional Factors, and Improved *In Vitro* Digestion Properties

**DOI:** 10.3390/foods15213796

**Published:** 2026-10-23

**Authors:** Shiji Zhao, Zixian He, Xinyu Jiang, Bowen Tan, Huilin Li, Wei Ma, Wendi Fan, Baojie Jiang

**Affiliations:** 1 College of Science and Technology, Hebei Agricultural University, Cangzhou 061100, China; 2 College of Food Science and Pharmaceutical Engineering, Zaozhuang University, Zaozhuang 277160, China

**Keywords:** wheat bran valorization, solid-state fermentation, *Wolfiporia cocos*, mycotoxin reduction, bioactive compound release, *in vitro* digestion

## Abstract

To improve the comprehensive utilization value of wheat bran, solid-state fermentation (SSF) with *Wolfiporia cocos* was applied for biological modification. The fermentation conditions were optimized by single-factor tests and response surface methodology, and the optimal parameters were 5.20% inoculation amount, a solid–liquid ratio of 1:2.35, and fermentation at 28 °C for 17 days, yielding a FRAP value of 115.20 μmol/g. Unfermented wheat bran (CG) and fermented wheat bran (FWB) were then compared in proximate composition, phytic acid, total phenolics, flavonoids, and triterpenoids, and *in vitro* simulated digestion properties. After fermentation, the crude protein content increased by 16.0% and the water-soluble dietary fiber content increased by 102.6%, while the phytic acid content decreased by 18.0%. The extractable contents of total phenolics, flavonoids, and triterpenoids increased by 40.53%, 188.55%, and 28.86%, respectively. *In vitro* digestion showed that FWB released more nutrients and bioactive compounds in the intestinal phase than CG, and protein digestibility increased from 55.5% to 74.2%. The antioxidant capacity of the digestive fluid was also enhanced. Correlation analysis suggested this enhancement was associated with the release of phenolics and flavonoids; however, this relationship should be considered exploratory and requires further confirmation. In summary, *Wolfiporia cocos* fermentation modifies the nutritional composition of wheat bran, reduces anti-nutritional factors, and facilitates the release of active substances, supporting the valorization of wheat bran as a functional food ingredient and contributing to a more circular utilization of this low-value milling by-product.

## 1. Introduction

As one of the traditional grains in China, wheat has always ranked among the top in the world in terms of output. According to the National Bureau of Statistics of China, the total wheat output in 2024 reached 140.099 million tons. Wheat is usually processed into various foods, and wheat bran (WB), which accounts for 15–20% of the wheat grain, is a main by-product of wheat processing and represents a large and underutilized biomass resource [1]. Compared with other grain by-products, WB has a higher concentration of flavonoids, phytosterols and other secondary metabolites [2]. In particular, the relatively high concentration of polyphenols endows wheat bran with a stronger antioxidant capacity. Moreover, WB is rich in dietary fiber and minerals. Nevertheless, its nutritional value is adversely affected by a large number of anti-nutritional factors [3], which may lead to a decrease in feed intake, a slower growth rate, and a reduced feed conversion rate of livestock. On the other hand, with the growing public focus on healthy diets, natural raw materials rich in dietary fiber and polyphenols have attracted increasing attention in the development of functional foods. Although WB is abundant in beneficial components such as dietary fiber, phenolic acids and flavonoids [4], its application potential as an ingredient for functional foods has not been fully explored. Therefore, if appropriate processing technologies could be adopted to improve the proximate composition and digestive properties of WB and expand its application from a single feed raw material to a raw material applicable to functional foods or food ingredients, it would greatly enhance the comprehensive utilization value of wheat bran.

As an eco-friendly and mild processing strategy, solid-state fermentation (SSF) has been reported to improve the nutritional quality of cereal by-products, reduce anti-nutritional factor levels, and liberate bound bioactive compounds [5], thereby providing a feasible technical route for the valorization of wheat bran. *Wolfiporia cocos* (syn. *Poria cocos*) is a fungus that has long served as both food and medicine, and is commonly called “Fuling” in China. It is consumed not only in China but also in Japan and other Asian countries. In traditional Chinese medicine theory, this fungus is considered to exert spleen-strengthening, diuretic, sedative, and dampness-removing effects [6]. Such a long history of dietary and medicinal use provides a basis for considering *Wolfiporia cocos* fermentation as safe [7]. *Wolfiporia cocos* is rich in bioactive components, including β-(1→3)-D-glucans and tetracyclic triterpenoids, which are the dominant triterpenoid class in this fungus [7]. These compounds are considered the main chemical basis for the multiple physiological activities of *Wolfiporia cocos*, including anti-inflammatory and antioxidant activity [8], immunomodulation [9], and anti-tumor effects [10]. *Wolfiporia cocos* can secrete multiple extracellular enzyme systems, including cellulase and hemicellulase [11]. During its growth and metabolism, it disrupts the substrate cell wall structure and facilitates the release of bound bioactive compounds [12], thereby improving the nutritional value and functional properties of fermentation materials.

To better position *Wolfiporia cocos* among existing fermentation starters, it is useful to compare it with conventional strains used for wheat bran modification. Conventional solid-state fermentation strains for wheat bran modification include *Aspergillus* spp., *Rhizopus oryzae*, yeasts and lactic acid bacteria. Although *Aspergillus niger* exhibits favorable performance in some applications, it carries potential safety risks related to mycotoxin production [13]. *Rhizopus oryzae* can effectively degrade arabinoxylans and increase soluble dietary fiber in wheat bran, yet fermentation performance of *Rhizopus oryzae* is highly dependent on precise control of moisture content and incubation time [14]. Yeasts and lactic acid bacteria improve fermentation flavor and degrade phytate by secreting extracellular phytase [15]; nevertheless, their lignocellulose-degrading capacity is generally weaker than that of filamentous fungi [16]. In contrast, *Wolfiporia cocos* has a long history of use as both a traditional medicine and a food ingredient, and its safety has been documented in the *Chinese Pharmacopoeia* [17]. It can synthesize a complete lignocellulolytic enzyme system to degrade plant cell walls and facilitate the release of bioactive substances from substrates [18]. Meanwhile, characteristic bioactive metabolites such as triterpenoids and polysaccharides are synthesized alongside mycelial growth and accumulated in the fermentation matrix. Existing studies have demonstrated that solid-state fermentation with *Wolfiporia cocos* can markedly increase the contents of active ingredients and biological activities of various substrates. Wu et al. [19] found that fermented vine tea with *Wolfiporia cocos* has more flavonoids, tea polyphenols and polysaccharides. Similarly, the findings of Chen et al. [12] indicated that the biotransformation and bioaccessibility of active ingredients from *Radix Astragali* were increased by *Wolfiporia cocos* during solid-state fermentation and *in vitro* digestion and antioxidant activity evaluation.

Based on the known enzymatic repertoire and biosynthetic capabilities of *Wolfiporia cocos*, we hypothesized that solid-state fermentation of wheat bran with this fungus would: (1) secrete lignocellulolytic enzymes (cellulase, xylanase, β-glucosidase) that disrupt the wheat bran cell wall and release bound bioactive compounds; (2) produce phytase that degrades phytic acid, thereby reducing anti-nutritional factor levels; (3) synthesize triterpenoids de novo as secondary metabolites during mycelial growth, enriching the functional component profile of the fermented product; and (4) improve the *in vitro* digestibility of wheat bran protein and the bioaccessibility of bioactive compounds. To test this hypothesis, we optimized the fermentation conditions and systematically compared unfermented and fermented wheat bran in terms of proximate composition, anti-nutritional factors, bioactive compounds, and *in vitro* simulated digestion properties.

It should be noted that, compared with some fungal starters that accumulate high mycelial biomass, the protein increase achieved by *Wolfiporia cocos* fermentation may be moderate; however, the nutritional significance lies not only in the absolute increase but also in the enhanced protein digestibility and the simultaneous release of bioactive compounds, which together improve the overall utilization value of wheat bran.

This study aims to perform solid-state fermentation of wheat bran using *Wolfiporia cocos*, with antioxidant activity as the indicator to optimize fermentation process parameters. On this basis, the effects of fermentation on the proximate composition, anti-nutritional factors, functional bioactive compounds and *in vitro* digestive characteristics of wheat bran were systematically evaluated. The specific research contents are listed as follows: determining the optimal fermentation conditions through single-factor experiments and response surface methodology (RSM); analyzing the changes in proximate composition (crude protein, crude ash, and dietary fiber) before and after fermentation; investigating the degradation efficiency of phytic acid, a primary anti-nutritional factor; detecting the increased extractable content of functional bioactive substances including total phenols, flavonoids and triterpenoids; and exploring the changes in protein digestibility and release profiles of bioactive compounds of wheat bran in simulated gastrointestinal environments via *in vitro* simulated digestion assays. Through these multi-dimensional assessments, this study aims to clarify the changes in wheat bran quality induced by Wolfiporia cocos-mediated solid-state fermentation and to provide theoretical foundations and experimental evidence for the biotransformation of wheat bran from a low-value feed material into a functional food ingredient.

## 2. Materials and Methods

### 2.1. Materials

The *Wolfiporia cocos* used in this study was provided by the Shanxi Functional Food Research Institute, Shanxi Agricultural University (Taiyuan, Shanxi, China), and maintained in our laboratory under the accession number sxgny2018002. Wheat bran was obtained from Huainan, Anhui Province, China, and was a conventional milling by-product of common wheat (*Triticum aestivum* L.). The specific cultivar and detailed milling process information were not available from the supplier. Prior to fermentation, the wheat bran was dried to constant weight, ground, and passed through a 20-mesh sieve.

This study used the Total Antioxidant Capacity (T-AOC) Assay Kit and Cellulose Content Assay Kit (Beijing Box Shenggong Technology Co., Ltd., Beijing, China) and Phytic Acid Content Assay Kit (Beijing Solarbio Science & Technology Co., Ltd., Beijing, China). Petroleum ether, phenol, and perchloric acid were obtained from Sinopharm Chemical Reagent Co., Ltd. (Shanghai, China). Aluminum nitrate, potassium acetate, potassium ferricyanide, trichloroacetic acid, Folin–Ciocalteu reagent, sodium carbonate, 3,5-dinitrosalicylic acid, and citric acid were obtained from Tianjin Dongli District Tianda Chemical Reagent Factory (Tianjin, China). Glucose was supplied by Tianjin Zhiyuan Chemical Reagent Co., Ltd. (Tianjin, China). Vanillin, rutin, gallic acid, and L-ascorbic acid were purchased from Tianjin Fuchen Chemical Reagent Factory (Tianjin, China). Potassium sulfate was obtained from Tianjin Jiesen Chemical Co., Ltd. (Tianjin, China). Sodium chloride was purchased from Tianjin Guangfu Fine Chemical Research Institute (Tianjin, China). α-Amylase, pepsin, and pancreatin were purchased from Beijing Merit Biotechnology Co., Ltd. (Beijing, China). All chemicals were of analytical grade.

### 2.2. Inoculant and Fermentation

Frozen strains were revived on Potato Dextrose Agar (PDA) plates and incubated in a thermostatic incubator (HWS-150B, Tianjin Taisite Instrument Co., Ltd., Tianjin, China) at 28 °C until mycelia fully covered the plates (approximately 5 days), with two successive subcultures. Subculturing was performed under aseptic conditions using a sterilized surgical blade to cut the mycelium. Subsequently, 1 cm^2^ of activated mycelium was inoculated onto wheat bran plate medium (wheat bran:water = 1:1.5) and cultured under the same conditions until mycelia covered the plates (approximately 5 days).

**Figure 1:**
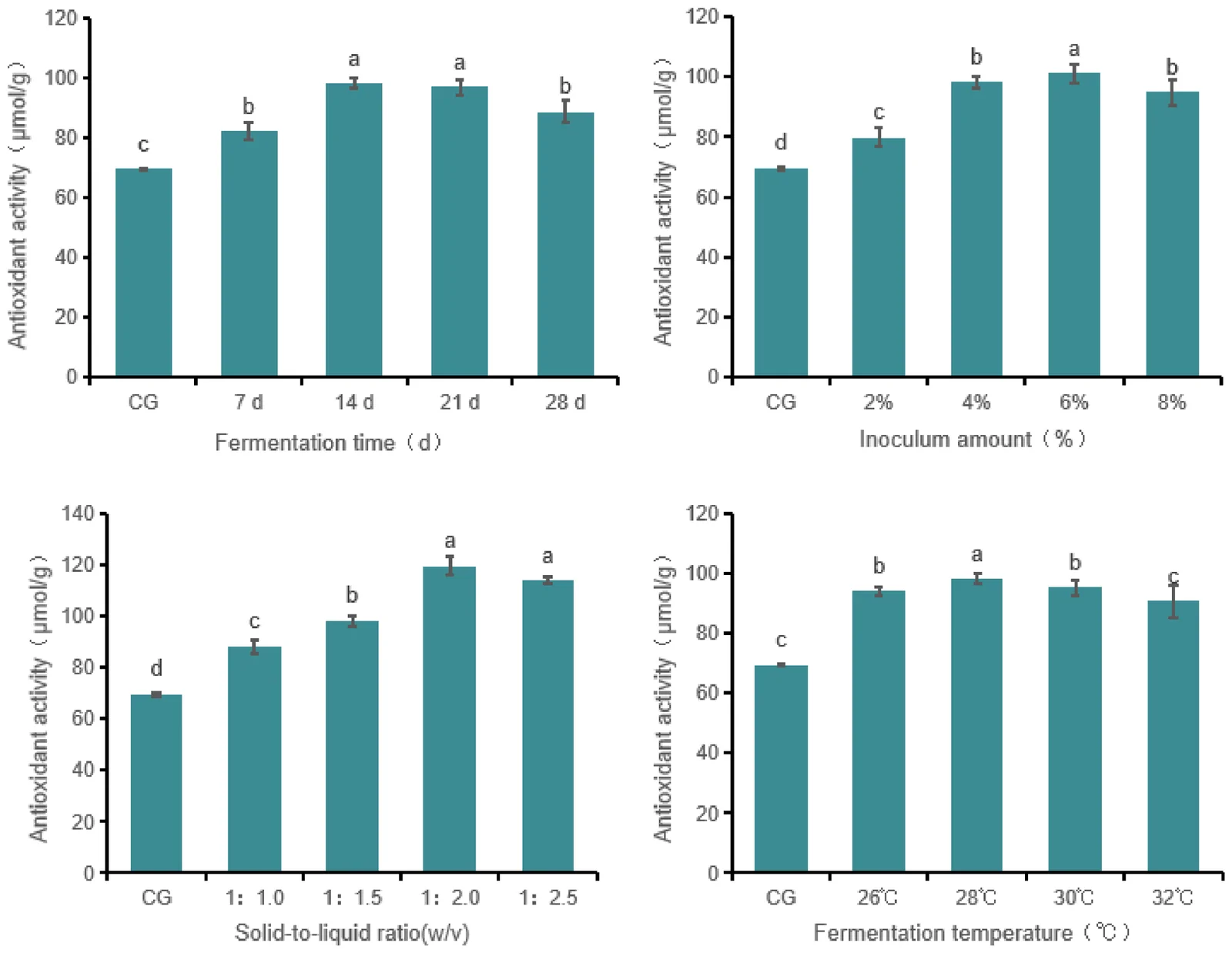
Single-factor experiments to analyze the effects of different factors on the antioxidant activity of *Wolfiporia cocos*-fermented wheat bran. Note: CG, unfermented wheat bran (control group); FWB, fermented wheat bran. Data are presented as mean ± SD (*n* = 3 biological replicates). Different superscript letters within a row indicate significant differences (*p* < 0.05).

WB SSF Medium: Briefly, 150 g WB and distilled water (1:2.5, *w*/*v*) were added into fermentation bags. The WB samples were autoclaved at 121 °C and 15 psi for 40 min. The inoculation amount was expressed as the number of mycelial plugs (1 cm^2^ each) per 100 g of dry wheat bran. The RSM-optimized inoculation amount was 5.20%, corresponding to 7.8 mycelial plugs per 150 g of dry wheat bran (equivalent to 5.2 mycelial plugs per 100 g of dry wheat bran). The seed culture was inoculated, and fermentation was carried out at 28 °C for 17 days. The fermentation time of 17 days was selected based on the results of single-factor experiments (Figure 1) and response surface optimization. Although this period is longer than that of conventional SSF starters, it was necessary to allow sufficient time for *Wolfiporia cocos* to synthesize triterpenoids and extensively degrade the wheat bran cell wall, which are the key advantages of this fermentation system. The control group (CG) was prepared under identical conditions, including the same solid–liquid ratio (1:2.5, *w*/*v*), autoclaving at 121 °C and 15 psi for 40 min, and incubation at 28 °C for 17 days, but without inoculation of *Wolfiporia cocos*. This design allowed the effect of fermentation to be isolated from the effects of moisture, sterilization, and incubation temperature. After fermentation, the culture substrate was dried at 50 °C, ground, and sieved through a 60-mesh sieve. The prepared samples were stored at 4 °C prior to analysis.

It should be noted that dry matter recovery after fermentation was not measured in this study. During solid-state fermentation, part of the substrate dry matter is consumed as a carbon and energy source for mycelial growth, and part may be lost through respiration and evaporation, resulting in dry matter loss. Because the primary aim of this study was to evaluate the effects of fermentation on nutritional composition, anti-nutritional factors, bioactive compounds, and *in vitro* digestion properties, dry matter recovery was not included in the analytical workflow. Therefore, the concentrations of components reported in this study are expressed on a dry-weight basis, and the possible contribution of the relative enrichment effect caused by dry matter loss should be considered when interpreting the results.

### 2.3. Experimental Design and Process Optimization

#### 2.3.1. Completely Randomized Design (CRD)

A completely randomized design (CRD) was adopted in this study to investigate the effects of four factors, namely inoculation amount, solid–liquid ratio, fermentation temperature and fermentation time, on the antioxidant activity of *Wolfiporia cocos*-fermented wheat bran. Four factors with four levels for each factor were arranged in independent random treatments.

#### 2.3.2. Response Surface Methodology (RSM)

Based on the results of the completely randomized design, three factors that exerted significant promoting effects on the antioxidant activity of *Wolfiporia cocos*-fermented wheat bran were identified for the Box–Behnken experimental design [20]. The Box–Behnken design, model fitting, analysis of variance (ANOVA), and residual diagnostics were performed using Design-Expert 13 (Stat-Ease, Inc., Minneapolis, MN, USA).

### 2.4. Analytical Methods

#### 2.4.1. Antioxidant Activity

Total reducing power was determined by the potassium ferricyanide assay. FRAP value was determined using assay kits. In vitro antioxidant capacity was determined by the DPPH assay.

Wheat bran samples (1.0 g) were mixed with 15 mL of 80% (*v*/*v*) ethanol at a sample-to-solvent ratio of 1:15 (*w*/*v*) and extracted in an ultrasonic bath at room temperature for 2 h. After centrifugation, the supernatant was collected and used for the DPPH and total reducing power assays.

DPPH assay. The reaction mixture contained 1.0 mL of sample extract and 3.0 mL of 0.1 mmol/L DPPH ethanol solution. After 30 min in the dark at room temperature, the absorbance was measured at 517 nm. The scavenging rate was calculated as *p* (%) = [1 − (As − Ac)/Ab] × 100.

Total reducing power. The assay was performed by the potassium ferricyanide method. Briefly, 1.0 mL of sample extract was mixed with phosphate buffer (pH 6.6) and 1% potassium ferricyanide, incubated at 50 °C for 20 min, and then treated with 10% trichloroacetic acid. After centrifugation, the supernatant was mixed with distilled water and 0.1% FeCl_3_, and the absorbance was measured at 700 nm. Vc was used as the standard (y = 0.0046x + 0.0608, R^2^ = 0.996).

For the FRAP assay, the sample was prepared using the commercial kit according to the manufacturer’s instructions. Approximately 0.1 g of sample was mixed with 1 mL of pre-cooled extraction buffer at a ratio of 1:10 (g:mL), homogenized in an ice bath, and centrifuged at 10,000 rpm for 10 min at 4 °C. The supernatant was collected and kept on ice until measurement.

#### 2.4.2. Proximate Composition

Crude protein. Crude protein content was determined by the Kjeldahl method according to GB 5009.5-2025 [21]. Briefly, approximately 0.5 g of dried sample was digested with 10 mL of concentrated sulfuric acid and a catalyst tablet at 420 °C for 1 h. After cooling, the digest was distilled with 40% (*w*/*v*) NaOH, and the released ammonia was collected in 2% (*w*/*v*) boric acid solution and titrated with 0.1 mol/L HCl. Crude protein content was calculated as total nitrogen × 6.31. It should be noted that crude protein was calculated as total nitrogen × 6.31. In fungal-fermented matrices, non-protein nitrogen sources such as chitin and nucleic acids may contribute to the total nitrogen, potentially leading to an overestimation of true protein content. Therefore, the crude protein values reported here should be interpreted as estimates rather than absolute protein contents.

Crude fat content was determined by the Soxhlet extraction method using petroleum ether as the solvent. Approximately 2 g of dried sample was extracted in a Soxhlet apparatus (Tianjin Tianke Glass Instrument Manufacturing Co., Ltd., Tianjin, China) in a water bath at 60 °C for 6 h. The solvent was then evaporated, and the residue was dried and weighed [22].

Crude ash. Crude ash content was determined according to GB 5009.4-2016 [23]. It was determined by incineration in a muffle furnace at 550 °C for 4 h until constant weight.

Moisture. Moisture content was determined using a rapid moisture analyzer (LSD-S35B, Xiamen Laiside Scientific Instrument Co., Ltd., Xiamen, China).

Cellulose and soluble dietary fiber. Cellulose and soluble dietary fiber contents were determined using commercial assay kits (Beijing Box Shenggong Technology Co., Ltd., Beijing, China) according to the manufacturer’s instructions.

All results are expressed on a dry-weight basis.

#### 2.4.3. Bioactive Compounds

Triterpenoid content was determined using the vanillin–acetic acid and perchloric acid colorimetric method [24]. The calibration curve was constructed using oleanolic acid as the standard:

y = 305.59x − 12.391 (R^2^ = 0.9958), where y is the absorbance, and x is the oleanolic acid concentration (mg/mL). Results were expressed as mg oleanolic acid equivalents per gram of dry weight (mg OAE/g DW).

The flavonoid content was determined by the aluminum nitrate–potassium acetate colorimetric method [25]. The calibration curve was constructed using rutin as the standard:

y = 0.1351x + 0.0451 (R^2^ = 0.999), where y is the absorbance, and x is the rutin concentration (mg/mL). Results were expressed as mg rutin equivalents per gram of dry weight (mg RE/g DW).

The total phenolic content was determined using the Folin–Ciocalteu method [26]. The calibration curve was constructed using gallic acid as the standard:

y = 0.0875x + 0.0574 (R^2^ = 0.997), where y is the absorbance, and x is the gallic acid concentration (mg/mL). Results were expressed as mg gallic acid equivalents per gram of dry weight (mg GAE/g DW).

The content of polysaccharides was determined using the phenol–sulfuric acid method [27]. The standard curve was established using glucose standards:

y = 0.0054x + 0.0385 (R^2^ = 0.999), where y is the absorbance, and x is the glucose concentration (mg/mL). Results were expressed as mg glucose equivalents per gram of dry weight (mg GE/g DW).

The determination of reducing sugars was performed according to Ma et al. [28]. The standard curve was established using glucose standards:

y = 0.483x − 0.021 (R^2^ = 0.994), where y is the absorbance, and x is the glucose concentration (mg/mL). Results were expressed as mg glucose equivalents per gram of dry weight (mg GE/g DW).

#### 2.4.4. Anti-Nutritional Factor

Phytic acid content was determined using a commercial assay kit (BC5845, Beijing Solarbio Science & Technology Co., Ltd., Beijing, China) according to the manufacturer’s instructions. Briefly, approximately 0.05 g of dried sample was mixed with 1 mL of extraction buffer I at a sample-to-solvent ratio of 1:5–20 (*w*/*v*). The mixture was homogenized and shaken at room temperature for 2 h, then centrifuged at 10,000× *g* for 10 min at 4 °C. An aliquot of 0.8 mL of the supernatant was mixed slowly with 0.15 mL of extraction buffer II, and the mixture was centrifuged again at 10,000× *g* for 10 min at 4 °C. The final supernatant was collected for analysis.

The assay is based on the enzymatic hydrolysis of sodium phytate by phytase to release inorganic phosphorus, which reacts with molybdate under acidic conditions to form a molybdenum blue complex with maximum absorbance at 660 nm.

#### 2.4.5. Mycotoxin Contamination

Indicators of fermented substrates were detected using commercial assay kits for ochratoxin A, aflatoxin B1, deoxynivalenol, and zearalenone, respectively. The biosafety of fermented products was evaluated in accordance with Chinese national food safety standards [29]. The total bacterial count and total mold count were determined referring to GB/T 13093-2023 [30] and GB 4789.15-2016 [31], respectively.

#### 2.4.6. *In Vitro* Digestion Simulation

A three-stage static *in vitro* digestion model (oral, gastric, and intestinal) was established according to the INFOGEST 2.0 protocol [32], with minor modifications.

Enzyme preparation. Salivary α-amylase solution was prepared by dissolving 15.0 mg of α-amylase in 20 mL physiological saline to give an activity of 750 U/mL. Pepsin solution was prepared by dissolving 2.60 g of pepsin in 20 mL of 0.02 mol/L HCl to give an activity of 2000 U/mL. Pancreatin solution was prepared by dissolving 19.0 g of pancreatin (8× USP) in 20.0 mL phosphate buffer. Enzyme activities were used as stated by the supplier and were not independently calibrated against the INFOGEST assay.

Oral phase. A 2 g portion of wheat bran sample was mixed with 4.0 mL physiological saline, and 1.0 mL of salivary α-amylase solution was added. The mixture was incubated at 37 °C for 5 min.

Gastric phase. The oral digest was mixed with 9.0 mL of 0.02 mol/L HCl, and the pH was adjusted to 2.0 with 1 mol/L HCl. Then, 1.0 mL of pepsin solution was added. The mixture was incubated at 37 °C for 2 h, with sampling every 30 min.

Intestinal phase. The gastric digest was mixed with 19.0 mL phosphate buffer, and the pH was adjusted to 6.8. After mixing, 1.0 mL of pancreatin solution was added, and the mixture was incubated at 37 °C for 2 h, with sampling every 30 min. Enzyme inactivation and sample collection. At each sampling point, enzymatic activity was quenched by heating in a 70 °C water bath for 3 min. Samples were then centrifuged (8000 rpm, 10 min), and the supernatants were collected and stored at 4 °C until analysis. The release of protein, triterpenoids, total phenolics, and flavonoids, as well as the antioxidant activity of the digestive fluids, were determined as described above. Protein digestibility was determined by the trichloroacetic acid precipitation method.

### 2.5. Statistical Analysis

All experiments were performed with three biological replicates (*n* = 3). For the optimization experiments (single-factor tests and response surface methodology), each treatment was performed with three biological replicates, and analysis of variance (ANOVA) combined with multiple comparisons was used for significance testing. Data are presented as mean ± standard deviation (SD). The normality of data distribution was assessed using the Shapiro–Wilk test, and the homogeneity of variance was evaluated using Levene’s test. For comparisons between the control group (CG) and the fermented wheat bran group (FWB), an independent samples *t*-test was used when data met the assumptions of normality and homogeneity of variance; otherwise, Welch‘s *t*-test was applied. For multiple comparisons among more than two groups, one-way ANOVA followed by Tukey’s post hoc test was used when the assumptions of normality and homogeneity of variance were met. When variances were unequal, Welch‘s ANOVA with the Games–Howell post hoc test was applied. When data did not meet the normality assumption, the Kruskal–Wallis test followed by Dunn’s post hoc test was used. For all statistical tests described above, statistical significance was set at *p* < 0.05. Pearson correlation analysis was performed to evaluate the relationship between the release rates of bioactive compounds and antioxidant activity during *in vitro* simulated digestion, with significance also set at *p* < 0.05. All statistical analyses were performed using SPSS Statistics 26.0 (IBM Corp., Armonk, NY, USA). Graphical visualization was performed using Origin 2022 (OriginLab Corp., Northampton, MA, USA).

## 3. Results and Analysis

### 3.1. Results of SSF Process Optimization

**Figure 2:**
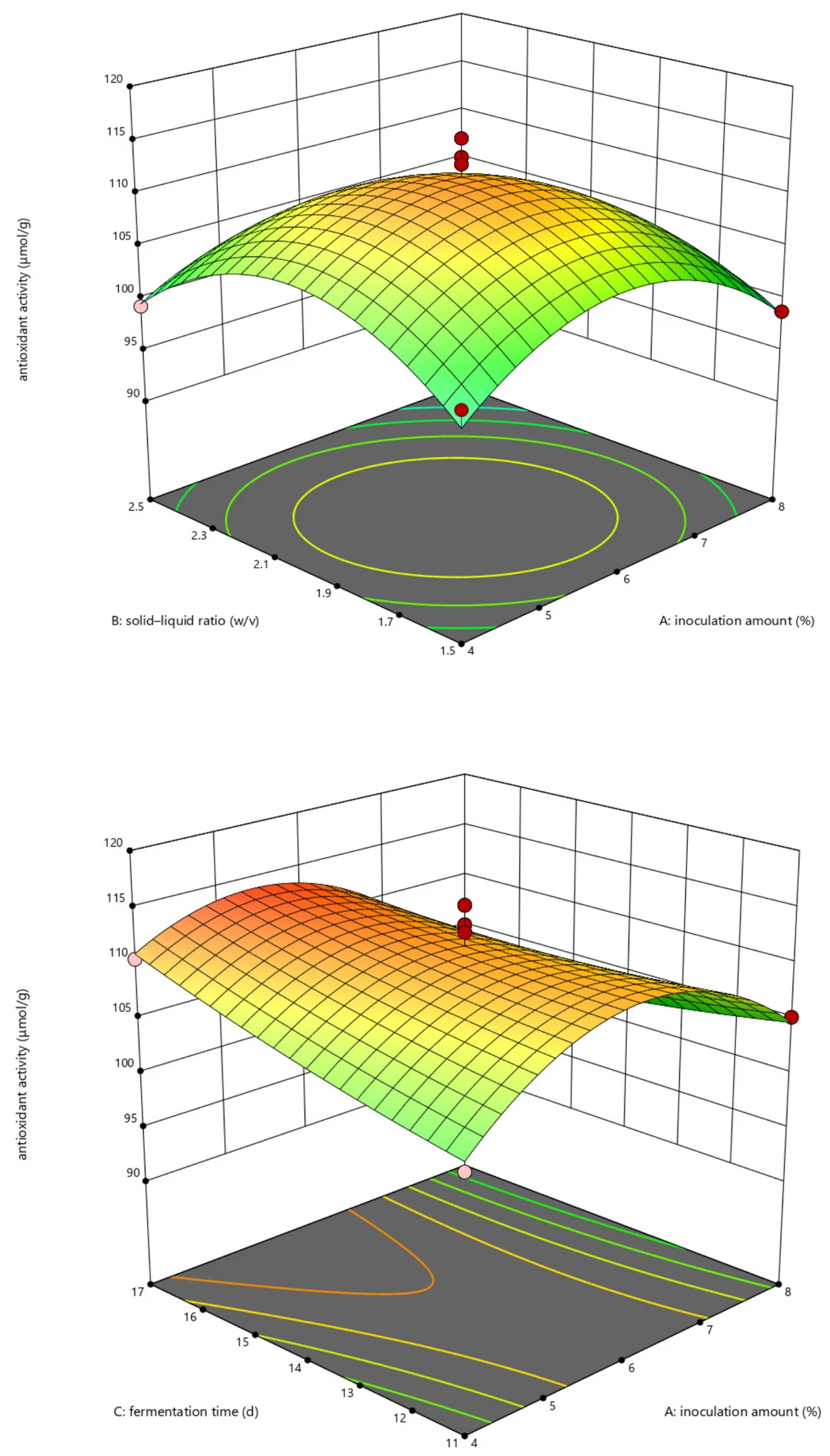

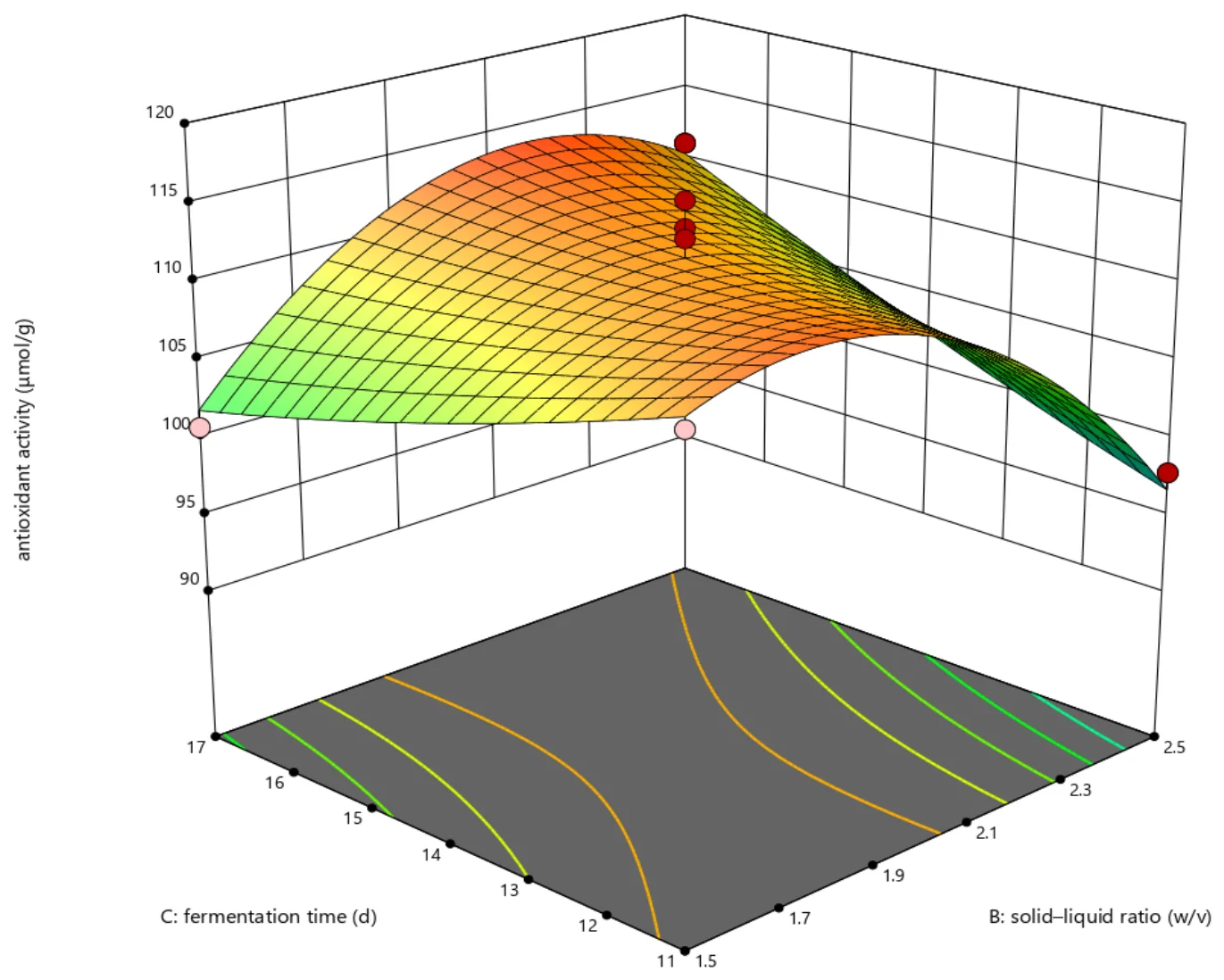
Three-dimensional response surface plots showing the interactive effects of fermentation time, solid–liquid ratio, and inoculum amount on the antioxidant activity of fermented products. Note: A = inoculation amount (%), B = solid–liquid ratio (*w*/*v*), and C = fermentation time (d).

Figure 1 illustrates the changes in antioxidant activity of *Wolfiporia cocos*-fermented wheat bran under different conditions. Inoculum size, fermentation time, and solid–liquid ratio significantly enhanced antioxidant capacity, with optimal single-factor levels of 6%, 14 d, and 1:2.0, respectively, and an optimal temperature of 28 °C. These three factors were selected for RSM (Table 1), and three-dimensional response surface plots (Figure 2) were generated to evaluate their pairwise interactions. The regression model predicted optimal process parameters as an inoculum size of 5.24%, a solid–liquid ratio of 1:2.35, and a fermentation time of 16.97 d; these were adjusted to 5.20%, 1:2.35, 17 d, and 28 °C for practical applicability. Validation experiments yielded a mean FRAP of 115.20 ± 3.12 μmol/g, which deviated by only 0.44% from the predicted value and was 66.7% higher than that of the unfermented raw material (69.09 μmol/g). These results confirm the reliability and predictive accuracy of the fitted model.

**Figure 3:**
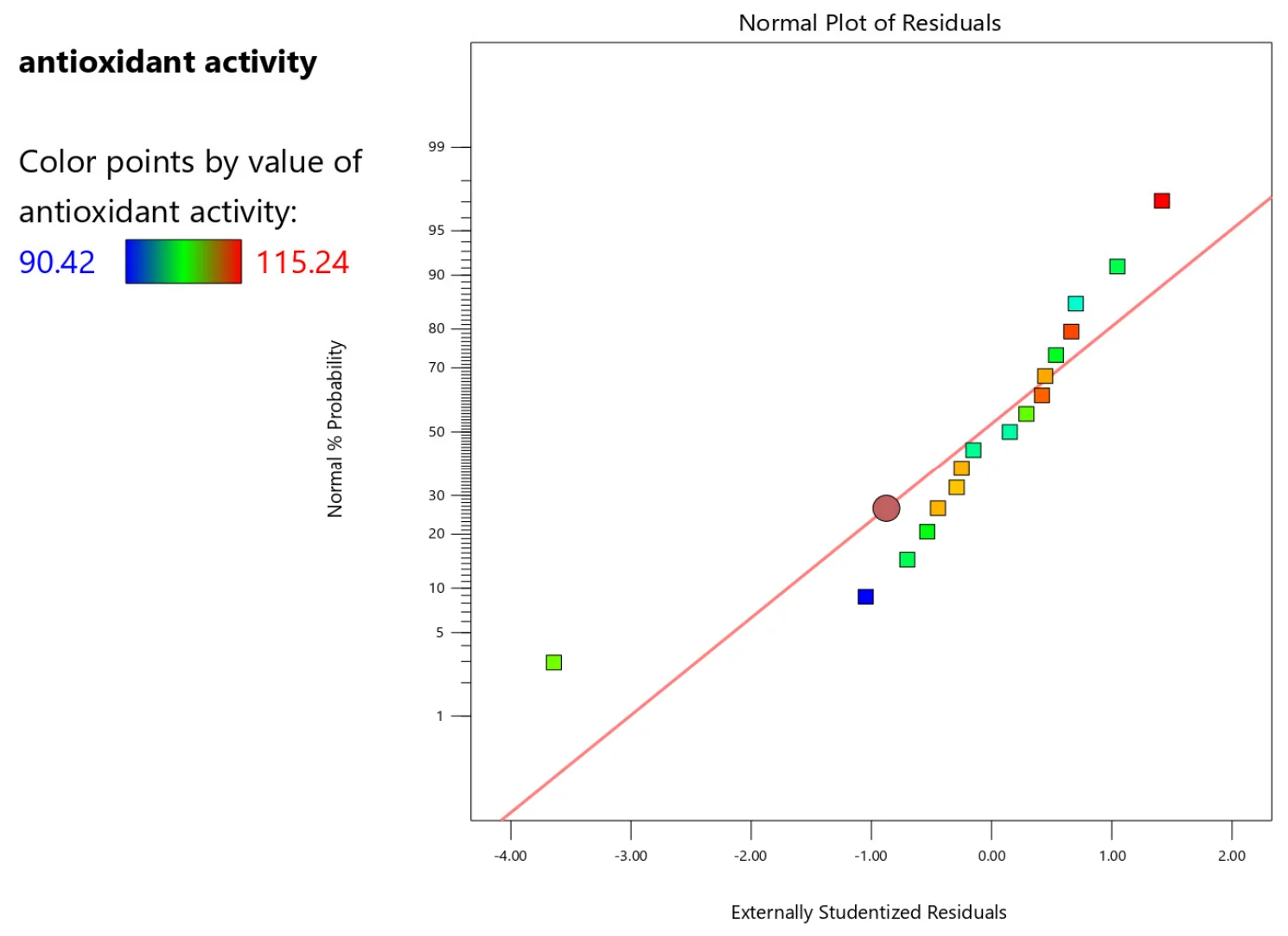
Normal probability plot of residuals for the antioxidant activity (FRAP) response. The data points fall approximately along a straight line, indicating that the residuals are approximately normally distributed and confirming the adequacy of the response surface model. Note: The red line represents the expected normal distribution line. Square symbols denote regular experimental points, and the circular symbol represents the central point of the Box–Behnken design. The color gradient of the squares corresponds to the antioxidant activity value, ranging from 90.42 (blue) to 115.24 (red).

The regression model for antioxidant activity was statistically significant (*p* = 0.0054), while the lack-of-fit test was not significant (*p* = 0.8518), indicating that the model adequately fitted the experimental data. The analysis of variance (ANOVA) for the fitted quadratic polynomial model is presented in Table 2. The coefficient of determination (*R*^2^) and adjusted coefficient of determination (*R*^2^*Adj*) were 0.9141 and 0.8037, respectively. The gap between *R*^2^ and *R*^2^Adj is mainly attributable to the inclusion of non-significant terms in the full quadratic model, rather than to overfitting, as confirmed by the non-significant lack-of-fit test. The coefficient of variation (CV) was 2.90%, and the adequate precision was 8.3808 (>4), further confirming the adequacy of the model. The normal probability plot of residuals showed that the data points fell approximately along a straight line (Figure 3), indicating that the residuals were approximately normally distributed. The fitted quadratic polynomial regression equation in coded terms was: Y = 111.622 − 2.08125A − 1.5875B + 1.00375C − 1.7AB − 2.8125AC + 5.845BC − 7.18725A^2^ − 7.05975B^2^ + 0.59275C^2^, where Y is the FRAP value (μmol/g); and A, B, and C are the coded values of inoculation amount (%), solid–liquid ratio (*w*/*v*), and fermentation time (d), respectively. The negative coefficients of A^2^, B^2^, AB, and AC indicate that the response surface has a maximum within the experimental range, consistent with the convex shape of the three-dimensional plots in Figure 2.

### 3.2. Antioxidant Activity

To verify the antioxidant performance of the fermented product under the optimized process conditions, three methods—DPPH radical scavenging rate, ferric reducing antioxidant power (FRAP), and total reducing power—were employed to comprehensively evaluate the antioxidant activity of wheat bran before and after fermentation; the results are presented in Table 3.

As shown in Table 3, the DPPH radical scavenging rate of the unfermented wheat bran (control group) was 37.00%, which significantly increased to 58.96% after *Wolfiporia cocos* solid-state fermentation, representing a 59.35% improvement (*p* < 0.05). The FRAP value increased from 69.09 μmol/g to 115.20 μmol/g, a 66.71% enhancement (*p* < 0.05). The total reducing power rose from 6.08 μmol/g to 58.20 μmol/g, an approximately 9.57-fold increase. These results indicate that *Wolfiporia cocos* solid-state fermentation under optimal conditions can significantly enhance the antioxidant activity of wheat bran.

### 3.3. Proximate Composition

As shown in Table 4, compared with unfermented wheat bran, the proximate composition of wheat bran underwent significant changes after *Wolfiporia cocos* solid-state fermentation. The crude protein content significantly increased from 195.22 mg/g DW to 226.54 mg/g DW (*p* < 0.05), representing a 16.0% improvement. The increase in crude protein content reflects two distinct contributions: first, the accumulation of *Wolfiporia cocos* mycelial protein synthesized during growth and metabolism [33]; second, the relative increase in extractable content of nitrogenous compounds due to carbohydrate consumption, which reduces the total substrate mass. Because fungal biomass was not independently quantified, the relative contribution of these two sources could not be precisely separated. Nevertheless, the increase in crude protein content enhances the protein contribution rate of wheat bran and helps compensate for its low crude protein content when used as a food raw material. The crude ash content increased from 42.33 mg/g DW to 52.22 mg/g DW, representing a 23.4% increase. Crude ash primarily reflects the total amount of mineral elements (e.g., calcium, phosphorus, potassium, magnesium) in the material. Its elevation may be attributed to the relative increase in extractable content of minerals following the metabolic consumption of organic matter in the substrate, rather than to an absolute increase in mineral content. Further studies are needed to determine the changes in specific mineral elements.

The water-soluble dietary fiber (WSDF) content significantly increased from 43.20 mg/g DW to 87.53 mg/g DW (*p* < 0.05), with an increase of 102.6%, making it the indicator with the largest variation among all selected chemical components. The substantial rise in WSDF originates from the hydrolysis of insoluble dietary fiber in wheat bran cell walls by cellulase and xylanase secreted by *Wolfiporia cocos*, converting it into soluble fragments [34]. The cellulose content did not change significantly (*p* > 0.05), indicating that *Wolfiporia cocos* exhibits a more prominent selective hydrolysis effect on hemicellulose, while its degradation of crystalline cellulose is limited. WSDF possesses favorable water-holding capacity, thickening properties, and gel-forming ability [35]. After ingestion, it can delay gastric emptying and increase satiety [36]; meanwhile, as a fermentation substrate for gut microbiota, it can be utilized to produce short-chain fatty acids [37], thereby contributing to intestinal health. The significant increase in WSDF content enhances the functional value of wheat bran as a dietary fiber source.

The crude fat content slightly decreased from 2.43 mg/g DW to 1.95 mg/g DW, possibly because lipids were utilized by *Wolfiporia cocos* as a carbon source during fermentation. The moisture content did not change significantly (*p* > 0.05). It should be noted that both CG and FWB samples were prepared with the same initial solid–liquid ratio (1:2.5, *w*/*v*) and fermented under identical conditions, except for the inoculation of *Wolfiporia cocos*. Therefore, the similar moisture content between CG and FWB indicates that the water balance was comparable in both groups under the tested fermentation conditions.

### 3.4. Anti-Nutritional Factor

Phytic acid is a major anti-nutritional factor in wheat bran, and its content directly affects the digestion and absorption efficiency of nutrients. In this study, the change in phytic acid content in wheat bran before and after *Wolfiporia cocos* solid-state fermentation was determined, and the results are presented in Table 5.

The phytic acid content showed an apparent decrease from 8.72 μmol/g DW to 7.15 μmol/g DW, corresponding to a reduction of 18.0% relative to the unfermented control. However, this difference should be interpreted as a trend rather than a statistically confirmed effect, given the relatively high standard deviation in the fermented group. The reduction in phytic acid is mainly attributed to phytase produced by *Wolfiporia cocos* during fermentation [38]. Since the endogenous phytase in wheat bran is inactivated by autoclaving, the degradation of phytic acid primarily results from the metabolic activity of *Wolfiporia cocos*. Phytic acid is the main storage form of phosphorus in wheat bran; however, humans and other monogastric animals lack endogenous phytase and thus cannot readily utilize the phosphorus bound in phytic acid. More importantly, the phytic acid molecule contains six phosphate groups and carries a strong negative charge under physiological conditions, exerting a potent chelating effect on divalent cations such as Ca^2+^, Fe^2+^, Zn^2+^, and Mg^2+^ [39]. This leads to the formation of insoluble phytate complexes, thereby substantially reducing the absorption and utilization of these mineral elements. Furthermore, phytic acid can form electrostatic complexes with protein molecules, inhibiting the hydrolytic action of proteases and further reducing protein digestibility [40]. The phytic acid content showed an apparent decrease after *Wolfiporia cocos* fermentation. Since phytic acid can chelate divalent cations and form complexes with proteins [41], a reduction in phytic acid could potentially alleviate these inhibitory effects. However, because this reduction did not reach statistical significance, a considerable amount of phytic acid remained after fermentation, and the bioavailability of minerals and proteins was not directly determined in this study; this interpretation should be considered tentative and requires further investigation. 

### 3.5. Bioactive Compounds

**Figure 4:**
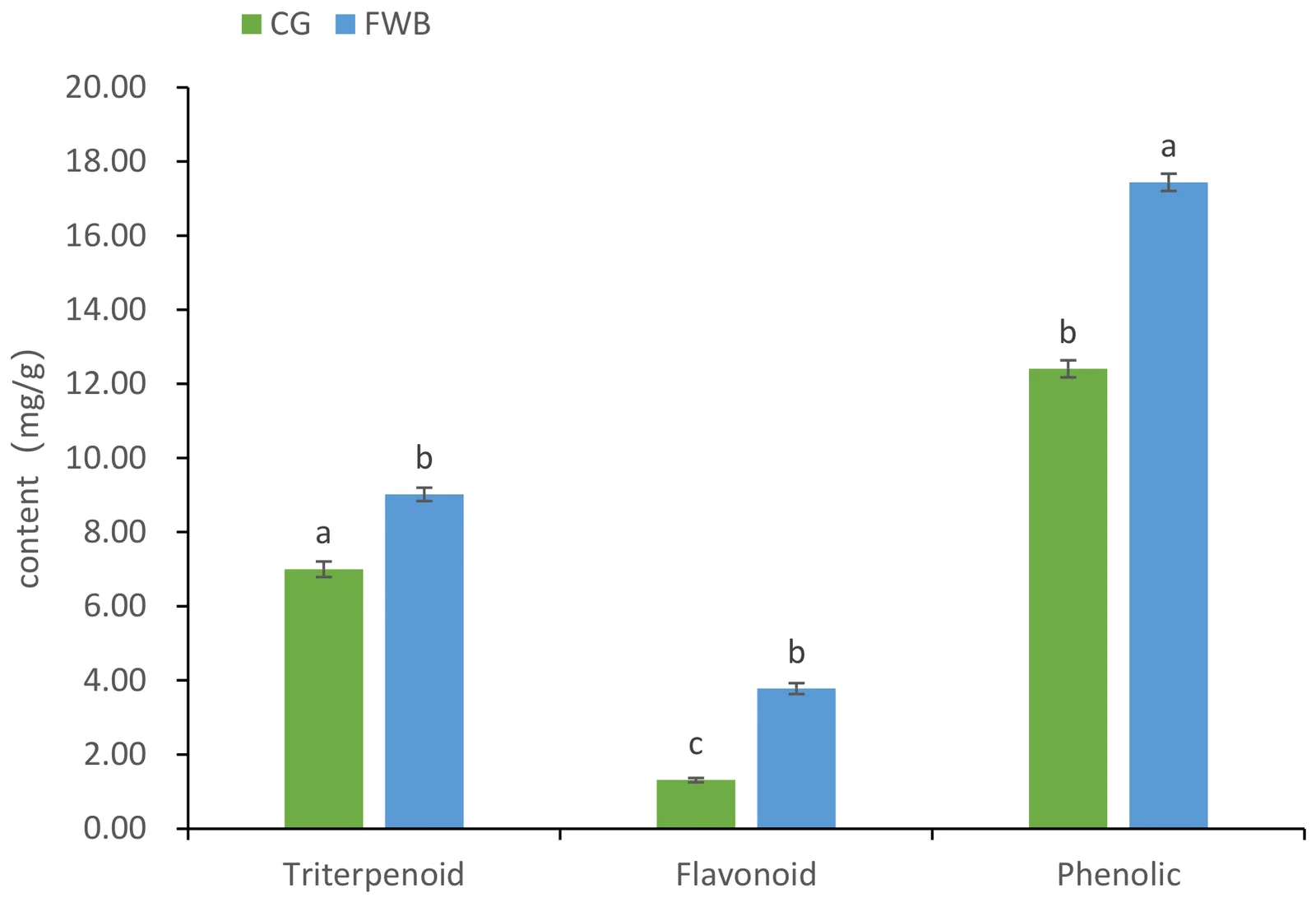
Variation in bioactive compound contents in fermented products. Note: Triterpenoids are expressed as oleanolic acid equivalents (mg OAE/g DW); flavonoids as rutin equivalents (mg RE/g DW); total phenolics as gallic acid equivalents (mg GAE/g DW). CG, unfermented wheat bran (control group); FWB, fermented wheat bran. Data are presented as mean ± SD (*n* = 3 biological replicates). Different superscript letters within a row indicate significant differences (*p* < 0.05).

Triterpenoids, flavonoids, and total phenolics are the main natural bioactive substances in wheat bran, possessing various physiological regulatory functions. In this study, the changes in the contents of these three active components in wheat bran before and after *Wolfiporia cocos* solid-state fermentation were determined, and the results are shown in Figure 4.

After fermentation, the triterpenoid content increased from 7.00 mg OAE/g DW to 9.02 mg OAE/g DW, representing a 28.86% increase based on the colorimetric assay. The increase in triterpenoids is predominantly derived from *Wolfiporia cocos* itself, as triterpenoids are characteristic secondary metabolites synthesized by this fungus during mycelial growth [42], and these substances are incorporated into the fermentation product along with mycelial growth. A minor contribution from the enzymatic release of bound triterpenoids in wheat bran cannot be excluded, but the fungal contribution is expected to dominate. Triterpenoids are among the main active components of *Wolfiporia cocos*, exhibiting various biological activities such as anti-inflammatory and immunomodulatory effects [7]. The increased triterpenoid content after fermentation further enriches the functional component profile of wheat bran.

The total phenolic content (TPC), expressed as gallic acid equivalents, increased from 12.41 mg GAE/g DW to 17.44 mg GAE/g DW, representing a 40.53% increase based on the colorimetric assay. However, it should be noted that the Folin–Ciocalteu assay is not specific for phenolic compounds and measures the total reducing capacity of the extract; different phenolic compounds may also exhibit different responses to the reagent. Therefore, the observed increase in TPC should be interpreted as an increase in total Folin-reactive reducing compounds rather than a definitive increase in phenolic compounds. Two possible explanations are proposed: first, extracellular enzymes secreted by *Wolfiporia cocos*, such as cellulase and pectinase, may disrupt the cell wall structure of wheat bran, releasing phenolic substances originally present in bound form into the free form [34]; second, microbial metabolic activity may degrade high-molecular-weight polyphenol polymers into low-molecular-weight phenolic monomers [11]. Both mechanisms could contribute to the increased Folin-reactive reducing capacity.

The total flavonoid content, expressed as rutin equivalents, showed an apparent increase from 1.31 mg RE/g DW to 3.78 mg RE/g DW, representing a 188.55% increase based on the colorimetric assay. Flavonoids in wheat bran mainly exist in glycosidic form, tightly bound to cell wall polysaccharides [43]. Two mechanisms may plausibly contribute to the apparent increase in total flavonoids. On the one hand, *Wolfiporia cocos* secretes cellulases, xylanases and β-glucosidase. Previous work has demonstrated that fermentation by *Wolfiporia cocos* markedly elevates β-glucosidase activity from 0.015 to 0.16 U/mL and cellulase activity from 0.03 to 0.11 U/mL [44], disrupting the cell-wall matrix and liberating bound flavonoids. This is consistent with a study on Monascus anka-fermented oats, in which phenolic release was strongly associated with the activities of carbohydrate-degrading enzymes [45]. On the other hand, fungal biotransformation may convert flavonoid glycosides into aglycones, which are more readily detected by the colorimetric method used in the present study. HPLC-MS analysis of fermented corn silk further verified massive hydrolysis of flavonoid glycosides and accumulation of aglycones, including luteolin [46]. It should be noted that, without compound-level profiling (e.g., HPLC or LC-MS), the relative contributions of these two mechanisms could not be precisely quantified. Nevertheless, because the colorimetric assay measures total flavonoids as rutin equivalents rather than individual compounds, and no compound-level profiling was performed, the observed increase cannot be definitively attributed to enzymatic release, biotransformation, or de novo synthesis, and should be interpreted as an apparent increase in total flavonoids. The well-documented enzymatic repertoire of *Wolfiporia cocos* suggests that enzymatic release and biotransformation are plausible contributors.

### 3.6. Mycotoxin Contamination

The contents of four major mycotoxins—ochratoxin A (OTA), aflatoxin B1 (AFB1), deoxynivalenol (DON), and zearalenone (ZEN)—in wheat bran before and after fermentation were determined. The mycotoxin panel was selected based on the known contamination patterns of wheat bran in China, where type B trichothecenes (particularly DON) and zearalenone are the predominant mycotoxins. Fumonisins are primarily associated with corn-based commodities, and T-2/HT-2 toxins, while possible, are less prevalent in wheat bran than type B trichothecenes [47]. Meanwhile, the total bacterial count (TBC) and total mold count (TMC) were examined. The results are presented in Table 6.

Regarding mycotoxins, the initial levels of OTA (25.06 μg/kg) and AFB1 (8.10 μg/kg) in wheat bran exceeded the maximum levels established by the European Union for unprocessed cereals [48]. After fermentation, the DON content decreased from 4.96 μg/kg to 2.20 μg/kg, OTA from 25.06 μg/kg to 21.93 μg/kg, and AFB1 from 8.10 μg/kg to 7.33 μg/kg, all showing varying degrees of decline. ZEN was below the detection limit in both groups. However, despite these reductions, the OTA and AFB1 levels in the fermented product remained above the EU food safety limits. These results indicate that solid-state fermentation with *Wolfiporia cocos* does not introduce new mycotoxin risk and can partially reduce pre-existing mycotoxin levels, but fermentation alone was not sufficient to bring the contaminated wheat bran into compliance with food safety standards. Further optimization of the fermentation process or additional decontamination steps would be required before the fermented product could be used as a functional food ingredient.

Regarding microbial indicators, the total bacterial count (TBC) decreased from 1.1 × 10^5^ CFU/g to 2 × 10^4^ CFU/g after fermentation, and the total mold count (TMC) decreased from 3 × 10^4^ CFU/g to 1 × 10^4^ CFU/g. The reduction in contaminating microorganisms may be related to *Wolfiporia cocos* becoming the dominant microbiota during fermentation, effectively inhibiting the growth of undesirable microbes through competitive inhibition and the antagonistic effects of its metabolites [49].

### 3.7. In Vitro Digestion Simulation

**Figure 5:**
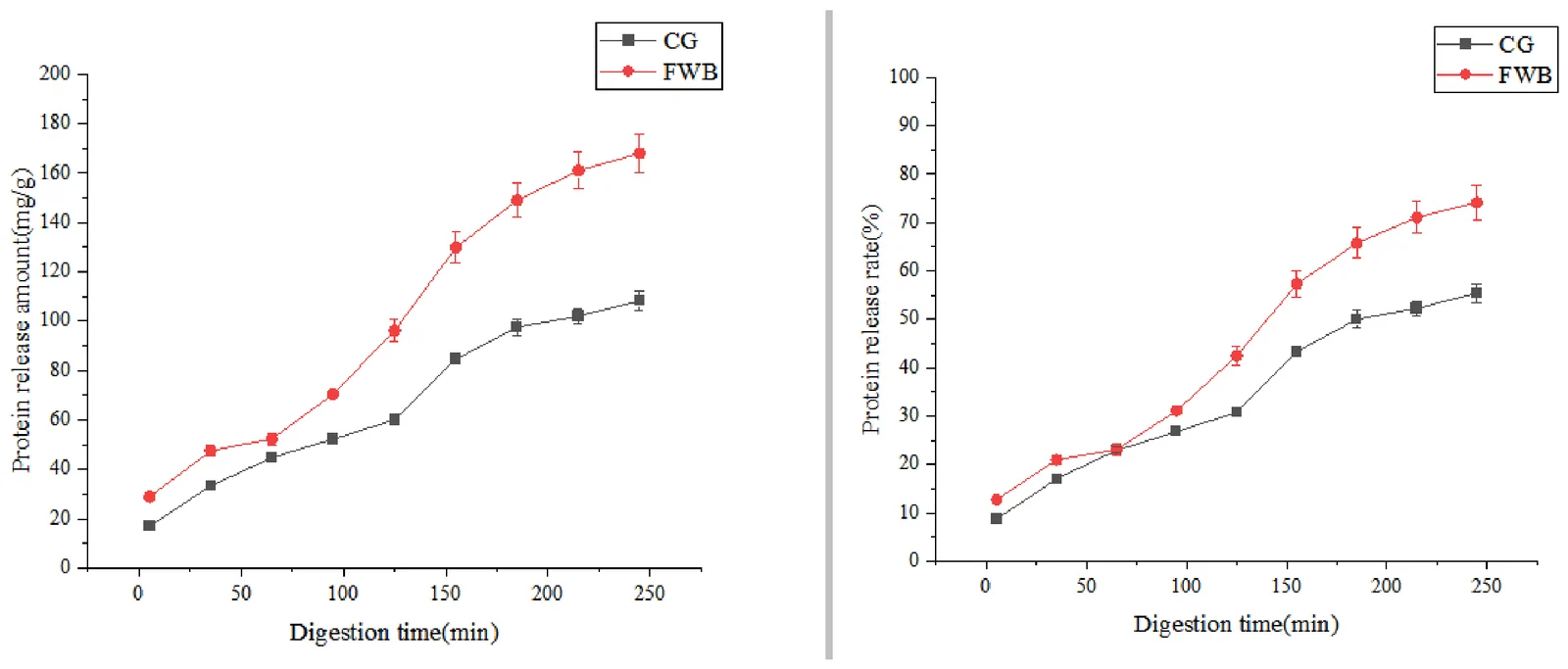
Dynamic release of proteins from fermentation products during simulated *in vitro* digestion. Note: CG, unfermented wheat bran (control group); FWB, fermented wheat bran. Data are presented as mean ± SD (*n* = 3 biological replicates).

**Figure 6:**
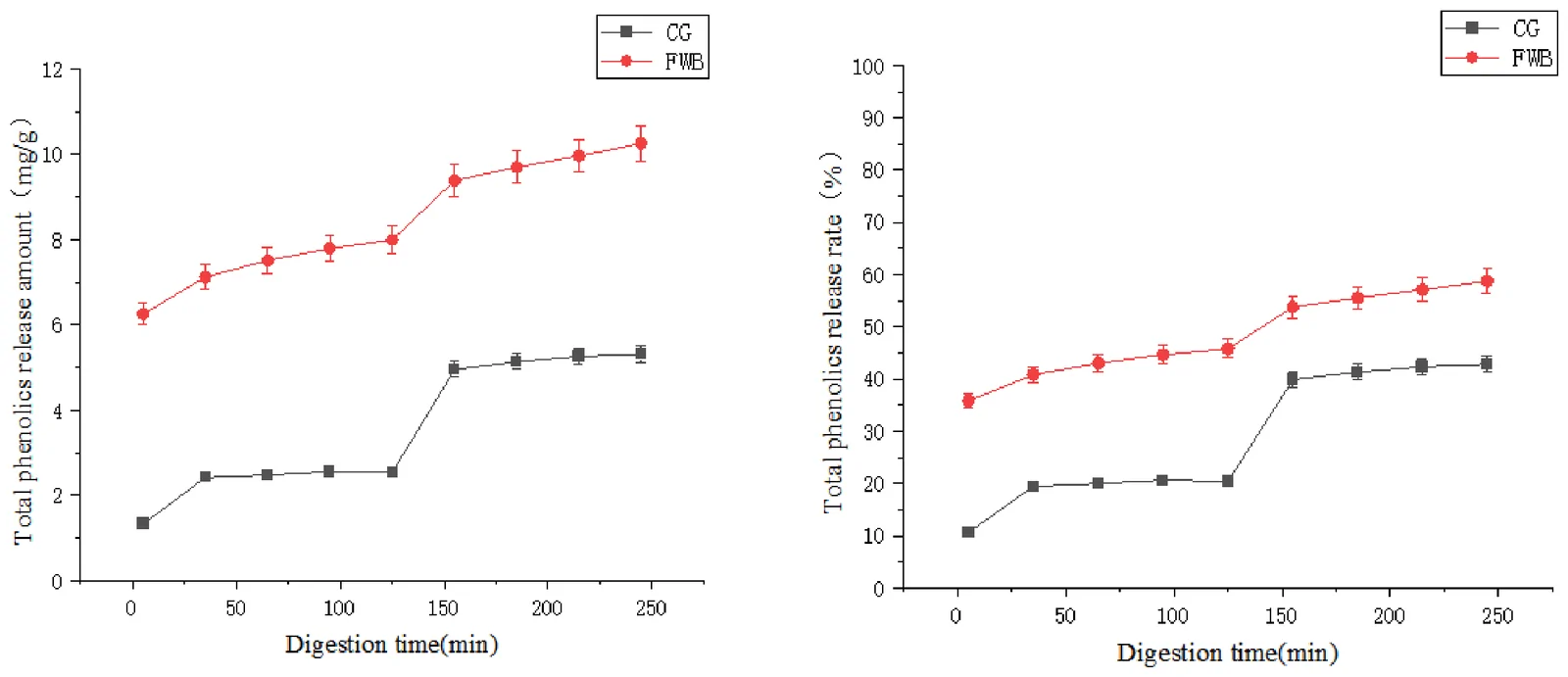
Dynamic release of total phenolics from fermentation products during simulated *in vitro* digestion. Note: CG, unfermented wheat bran (control group); FWB, fermented wheat bran. Data are presented as mean ± SD (*n* = 3 biological replicates).

**Figure 7:**
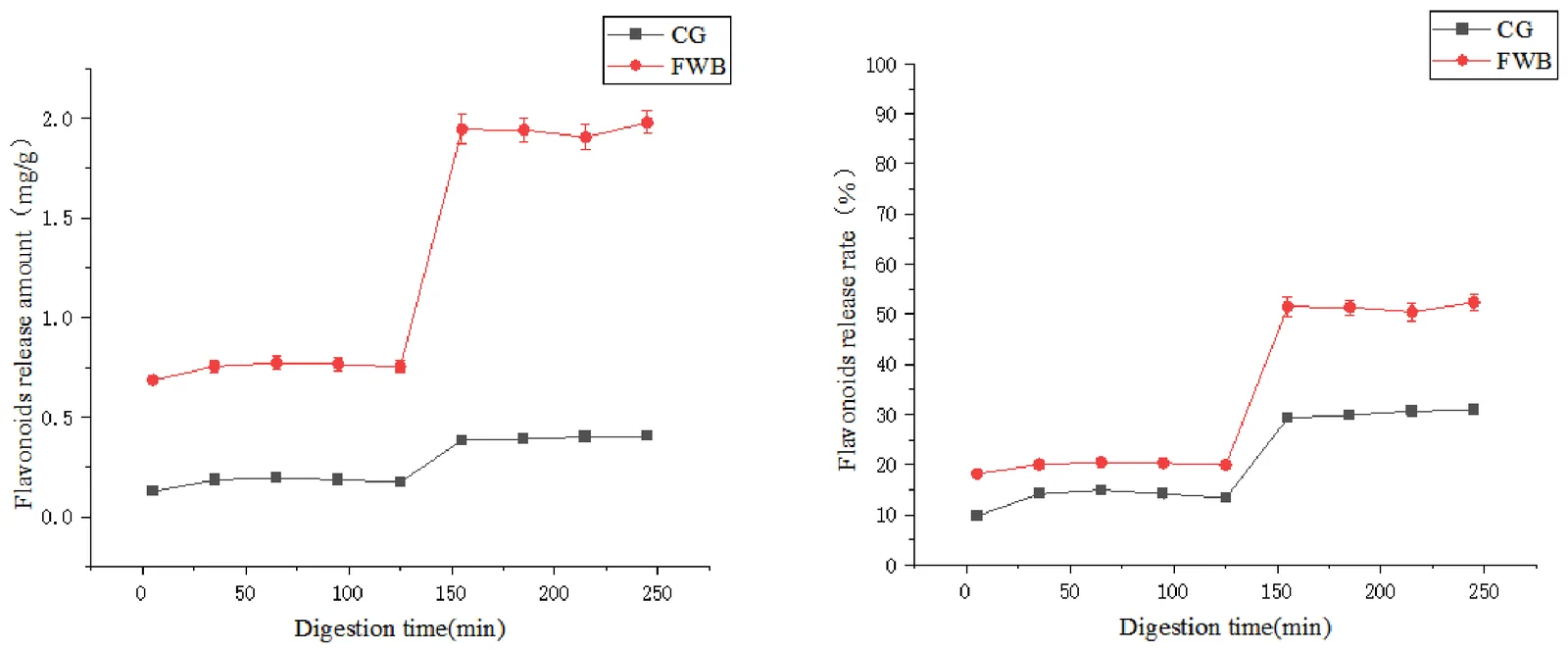
Dynamic release of flavonoids from fermentation products during simulated *in vitro* digestion. Note: CG, unfermented wheat bran (control group); FWB, fermented wheat bran. Data are presented as mean ± SD (*n* = 3 biological replicates).

**Figure 8:**
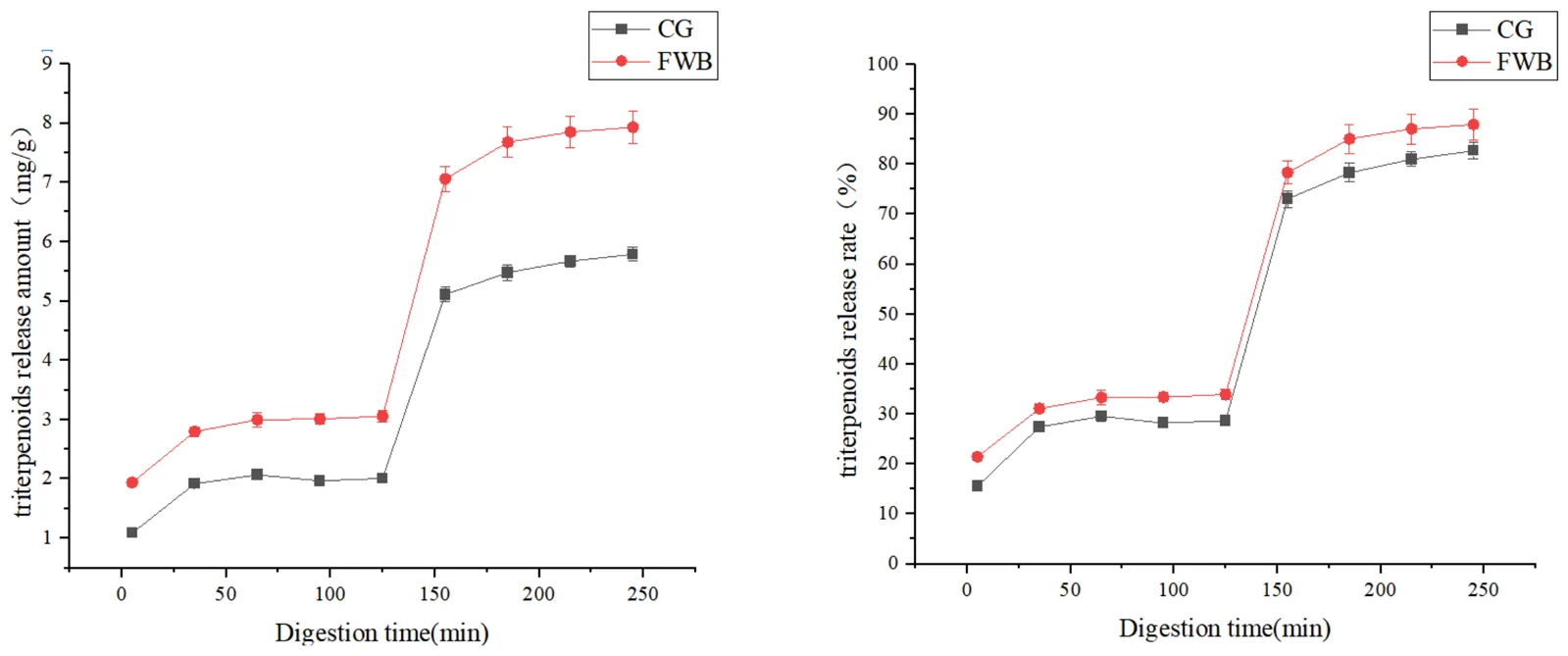
Dynamic release of triterpenoids from fermentation products during simulated *in vitro* digestion. Note: CG, unfermented wheat bran (control group); FWB, fermented wheat bran. Data are presented as mean ± SD (*n* = 3 biological replicates).

To evaluate the improvement effect of fermentation on the digestive properties of wheat bran, a three-stage *in vitro* simulated digestion model (oral–gastric–intestinal) was employed in this study. The protein digestibility and the release of total phenolics, flavonoids, and triterpenoids from wheat bran during digestion were determined before and after fermentation. Meanwhile, the antioxidant activity of the digestive fluid was measured. The results are shown in Figure 5, Figure 6, Figure 7 and Figure 8 and Table 7.

#### 3.7.1. Changes in Protein Digestibility

As shown in Figure 5, the protein digestibility increased from 55.5% to 74.2% (+33.7%) after fermentation. The improvement in protein digestibility mainly stems from two aspects: first, proteases secreted by *Wolfiporia cocos* during fermentation perform preliminary hydrolysis of wheat bran proteins, breaking down macromolecular proteins into peptide fragments that are more susceptible to digestive enzymes; second, fermentation disrupts the dense structure of the wheat bran cell wall, exposing proteins encapsulated within the cell wall and increasing their accessibility to digestive enzymes. Meanwhile, the degradation of phytic acid reduces interference with protease activity. The increase in protein digestibility means that after fermentation treatment, proteins in wheat bran are more readily broken down into amino acids and small peptides in the gastrointestinal environment, thereby enhancing the nutritional availability of protein. Jia et al. similarly reported that solid-state fermentation of wheat bran with edible fungal mycelium (*Pleurotus pulmonarius*) increased the crude protein content from 16.17% to 31.2% and improved the *in vitro* rumen crude protein digestibility from 78.8% to 96.38% [50], indicating that fungal fermentation can enhance the protein digestibility of plant-based materials.

#### 3.7.2. Changes in Bioactive Component Content

As shown in Figure 6, Figure 7 and Figure 8, the release amounts of total phenolics, flavonoids, and triterpenoids from fermented wheat bran during the intestinal phase were all significantly higher than those from unfermented wheat bran (*p* < 0.05). Specifically, the release of total phenolics increased by 389% compared with the raw material, flavonoids by 93%, and triterpenoids by 36%. For total phenolic quantification, a blank digestion without a wheat bran sample was run in parallel, and the Folin–Ciocalteu response of the blank was subtracted from all sample values to correct for interference from digestive enzymes and buffer components. It should be noted that the Folin–Ciocalteu method measures total reducing capacity rather than phenolics specifically, and may still react with sample-derived proteins and other reducing substances. Therefore, the absolute values should be interpreted as an estimate of total Folin-reactive compounds rather than strictly phenolic content. Nevertheless, because the same digestion protocol and blank correction were applied to both CG and FWB samples, the relative comparison between groups remains valid. The release of all three active components followed the trend of intestinal phase > gastric phase > oral phase, with the most pronounced release occurring during the intestinal phase. The increase in active component release is mainly attributed to the disruption of wheat bran cell wall structure by fermentation [34]. Total phenolics and flavonoids in wheat bran mainly exist in bound form within the cell wall, linked to structural polysaccharides such as cellulose and hemicellulose via ester bonds or glycosidic bonds. Cellulase and xylanase secreted by *Wolfiporia cocos* hydrolyze the cell wall framework, breaking the linkages between phenolic substances and polysaccharides and facilitating their dissolution during digestion. In contrast, triterpenoids showed a smaller increase in release, possibly because they are primarily located in the cytoplasm, with a distribution pattern distinct from that of cell wall-bound phenolics. The increased release of active components during simulated gastrointestinal digestion indicates that fermented wheat bran can release more functional bioactive substances in the gastrointestinal environment, which may contribute to their potential bioaccessibility.

#### 3.7.3. Changes in Antioxidant Activity of Digestive Fluid

Accompanying the dissolution of active components, the antioxidant activity of the digestive fluid also underwent significant changes. As shown in Table 7, after three-stage digestion, the DPPH radical scavenging rate, total reducing power, and total antioxidant capacity of the digestive fluid from fermented wheat bran were all significantly higher than those from the unfermented group (*p* < 0.05). Specifically, the total antioxidant capacity during the intestinal phase increased from 98.11 μmol/g to 164.65 μmol/g. Further Pearson correlation analysis indicated that the release of total phenolics and flavonoids was significantly positively correlated with total antioxidant capacity (total phenolics: r = 0.959–0.974, r^2^ = 0.920–0.949, *p* < 0.01; flavonoids: r = 0.850–0.973, r^2^ = 0.723–0.947, *p* < 0.05 or *p* < 0.01; *n* = 6). The corresponding Pearson correlation coefficients between bioactive compound release and antioxidant capacity are presented in Table 8. These correlations should be considered exploratory and hypothesis-generating, given the limited sample size (*n* = 6) and the absence of correction for multiple comparisons. An *in vitro* digestion study on fermented chrysanthemum rice wine [51] similarly found that 46.15% of metabolites were positively correlated with changes in DPPH and FRAP, consistent with the significant positive correlation between the release of total phenolics and flavonoids and antioxidant indicators observed in the present study. This finding suggests that the staged release of active components may contribute to the enhanced antioxidant activity of the digestive fluid. Further studies with larger sample sizes and compound-level identification are needed to confirm this relationship.

## 4. Discussion

Solid-state fermentation (SSF) is an effective approach to enhance the comprehensive utilization value of agricultural by-products, primarily through the biodegradation of the substrate cell wall by extracellular enzymes secreted by microorganisms [5]. In this study, *Wolfiporia cocos* was used as the fermentation strain to modify wheat bran via SSF. The fermented product showed changes in proximate composition, reduced anti-nutritional factor levels, enriched bioactive components, and enhanced *in vitro* digestion properties. It should be emphasized that the nutritional findings of this study should be interpreted as changes in nutritional composition rather than a comprehensive improvement in nutritional quality.

The modification of wheat bran by *Wolfiporia cocos* can be understood as an integrated sequence of biological events. First, *Wolfiporia cocos* secretes a broad spectrum of extracellular enzymes, including cellulase and xylanase [11,13], which hydrolyze the hemicellulose network of the wheat bran cell wall. This enzymatic degradation loosens the cell-wall matrix and reduces its physical barrier function. Second, cell-wall hydrolysis directly promotes the release of bound bioactive compounds. Phenolic acids and flavonoids in wheat bran are largely esterified or glycosidically linked to cell-wall polysaccharides, and their liberation is therefore tightly coupled to carbohydrate-degrading enzyme activity [43,44,45]. Third, the same enzymatic machinery contributes to the reduction in anti-nutritional factors. Phytase produced by *Wolfiporia cocos* degrades phytic acid [38], while disruption of the cell-wall matrix improves the accessibility of phytic acid to enzymatic hydrolysis. Fourth, and distinct from these degradative processes, *Wolfiporia cocos* synthesizes triterpenoids de novo as secondary metabolites during mycelial growth [43], incorporating them into the fermentation matrix. The result is a fermented product in which cell-wall hydrolysis, release of bound compounds, reduction in anti-nutritional factors, and de novo biosynthesis of triterpenoids occur simultaneously within a single food-grade fermentation step. This integrated mechanism, rather than any single isolated effect, is the basis of the functional improvement of fermented wheat bran.

Proximate composition. The crude protein content was calculated as total nitrogen × 6.31, and in fungal-fermented matrices, non-protein nitrogen sources such as chitin and nucleic acids may contribute to the total nitrogen, potentially leading to an overestimation of true protein content. Because fungal biomass was not independently quantified, the relative contributions of fungal mycelial protein and the relative enrichment effect caused by substrate dry matter loss could not be precisely separated. The 16.0% increase in crude protein concentration observed here is lower than the 93% increase reported for *Pleurotus pulmonarius*-fermented wheat bran [50]. However, that study targeted ruminant feed, and the protein increase was largely attributed to fungal mycelial biomass rather than modification of the bran matrix. In contrast, our system used a food-grade fungus and a shorter fermentation time, and the protein increase was accompanied by a 102.6% increase in water-soluble dietary fiber (WSDF). The value of this fermentation therefore lies in its multifactorial nature: it simultaneously improves fiber solubility, enriches bioactive compounds, reduces an anti-nutritional factor, and enhances protein digestibility. This combination is not commonly achieved by conventional SSF starters and represents the core novelty of the present work.

For WSDF, the increase reported here is comparable to the >120% increase achieved with *P. pulmonarius* [52] and higher than that reported for *Trichoderma reesei* and *Candida utilis* [16]. The selective increase in WSDF, with no significant change in cellulose, is consistent with the preferential hydrolysis of hemicellulose by cellulase and xylanase secreted by *Wolfiporia cocos* [34], as also reported for other fungal SSF systems [53]. Importantly, this increase may also enhance the prebiotic potential of fermented wheat bran. Wheat bran-derived arabinoxylan oligosaccharides have been shown to increase fecal bifidobacteria and short-chain fatty acid (SCFA) concentrations while lowering fecal pH in healthy adults [54]. Soluble dietary fiber isolated from fermented wheat bran has also been reported to up-regulate beneficial bacteria such as *Akkermansia* and increase acetic, propionic, and butyric acid levels in vivo [55]. Therefore, the enrichment of WSDF may improve the fermentability of wheat bran by gut microbiota and contribute to intestinal health. These findings suggest that the soluble fiber enrichment achieved here may have practical implications for gut health, although direct evidence from human or animal studies is still needed.

It should be noted that the increase in WSDF may affect the viscosity and rheological properties of wheat bran in aqueous systems. Depending on the specific processing conditions and intended technological application, such changes may be beneficial or detrimental. For example, a higher WSDF content may improve water-holding capacity and dough rheological properties [56], but may also increase viscosity and cause processing difficulties or undesirable texture in certain food systems [57]. Therefore, the processing performance of fermented wheat bran should be evaluated in the context of the specific food application, and no general claim of improved processing properties is made in this study.

The crude ash concentration increased by 23.4%, which likely reflects the relative enrichment of minerals after organic matter consumption rather than an absolute increase in mineral content. During fungal fermentation, organic matter (particularly carbohydrates) is consumed for mycelial growth, leading to a reduction in total substrate dry matter. As a result, the relative proportion of inorganic minerals increases even if the absolute mineral quantity remains unchanged. This mechanism is well documented: fungi convert 40–50% of carbohydrate carbon into CO_2_, causing organic matter loss that directly leads to ash enrichment [58]. Consistent with this, a recent study on Pleurotus ostreatus-fermented brewer‘s spent grain reported that ash content showed a strong positive correlation with substrate consumption, alongside significant negative correlations between carbohydrate/lipid contents and substrate consumption [59]. Therefore, the increase in crude ash should be interpreted as a relative concentration effect, and specific mineral elements were not determined in this study; direct measurement of individual minerals would be required to confirm whether any absolute changes occur.

The approximately 20% decrease in crude fat content is consistent with the metabolic activity of *Wolfiporia cocos* during solid-state fermentation. Fungi can utilize lipids as a carbon and energy source, particularly under conditions where readily fermentable carbohydrates become limiting. Lipid turnover by fungi in solid-state fermentation systems has been documented [60], with lipids being consumed after carbon source depletion. The slight reduction in crude fat observed here likely reflects partial utilization of wheat bran lipids by *Wolfiporia cocos* during the 17-day fermentation period. Although the absolute decrease is small (from 2.43 to 1.95 mg/g DW), it is consistent with fungal metabolic activity rather than indicating a substantial loss of nutritional value.

Finally, total carbohydrate, starch, arabinoxylan, and monosaccharide composition were not determined, and therefore a complete carbohydrate balance could not be established. During fungal fermentation, part of the substrate dry matter is consumed as a carbon and energy source for mycelial growth, resulting in dry matter loss. As a consequence, the concentrations of some components may increase partly due to the relative enrichment effect rather than net synthesis. Although dry matter recovery was not measured in this study, the observed increases in crude protein, crude ash, and WSDF should be interpreted with this consideration in mind.

Anti-nutritional factors. The phytic acid content showed an apparent decrease from 8.72 μmol/g DW to 7.15 μmol/g DW after *Wolfiporia cocos* SSF, corresponding to an 18.0% reduction. However, this difference did not reach statistical significance, and the relatively high standard deviation in the fermented group indicates considerable variability. Therefore, this reduction should be interpreted as a trend rather than a confirmed effect. This apparent reduction is lower than the approximately 37% degradation reported for lactic acid bacteria fermentation [16]. However, lactic acid bacteria lack the lignocellulolytic enzyme repertoire of filamentous fungi and do not produce triterpenoids. In pulse solid-state fermentation with *Bacillus subtilis*, phytic acid degradation occurred early during fermentation and coincided with maintained or improved amino acid digestibility [61], confirming that phytic acid reduction is a common outcome of solid-state fermentation across different substrates and microbial systems. Compared with these systems, the apparent phytic acid reduction achieved here is moderate but occurs simultaneously with cell wall degradation and enhanced release of bioactive compounds, which is not typically observed in single-strain bacterial fermentations. Since a considerable amount of phytic acid remained after fermentation, and the bioavailability of minerals and proteins was not directly determined, the main benefit of *Wolfiporia cocos* fermentation should not be attributed to phytic acid reduction alone, but rather to the combined effects on dietary fiber solubilization and bioactive compound release.

Bioactive components. The 40.53% apparent increase in total phenolics, 188.55% apparent increase in flavonoids, and 28.86% apparent increase in triterpenoids observed here differ from the patterns reported for other SSF systems. Monascus anka-fermented oats showed a strong increase in phenolic release associated with carbohydrate-degrading enzyme activity [45], whereas *Aspergillus niger*-fermented corn silk showed extensive hydrolysis of flavonoid glycosides and accumulation of aglycones [46]. Clostridium butyricum-fermented wheat bran also increased phenolic content, but the increase was accompanied by changes in microstructure and nutrient release rather than enhanced release of triterpenoids [43]. The simultaneous increase in extractable phenolics, flavonoids, and triterpenoids observed here is consistent with the dual role of *Wolfiporia cocos*: it secretes β-glucosidase and other carbohydrate-degrading enzymes that release bound phenolics and flavonoids [11], and it synthesizes triterpenoids as secondary metabolites during mycelial growth [42]. This combination is not commonly reported for other SSF starters. It should be emphasized that these values are based on colorimetric quantification, which measures total equivalents rather than individual compounds, and the observed increases should therefore be interpreted as apparent increases in total colorimetric equivalents rather than confirmed increases in specific bioactive compounds.

Improvement of *in vitro* digestive properties. The increases in the release of total phenolics (389%), flavonoids (93%), and triterpenoids (36%) during the intestinal phase, together with the increase in protein digestibility from 55.5% to 74.2%, are consistent with previous studies on fungal-fermented plant materials. Asensio-Grau et al. [62] reported improved protein digestibility in lentil flour fermented with Pleurotus ostreatus, and Zhang et al. [51] found that fermentation enhanced the bioaccessibility of phenolic compounds in chrysanthemum rice wine. The present results show that *Wolfiporia cocos* fermentation simultaneously improves protein digestibility and the release of multiple classes of bioactive compounds.

Taken together, the comparison with previous studies suggests that the advantage of *Wolfiporia cocos* lies not in maximizing any single metric, but in simultaneously achieving dietary fiber solubilization, an apparent reduction in anti-nutritional factors, enhanced bioactive release (including triterpenoids), and enhanced *in vitro* protein digestibility within a single food-grade fermentation step. *P. pulmonarius* achieves higher protein increases but targets feed applications [50]; lactic acid bacteria degrade more phytic acid but lack lignocellulolytic enzymes [16]; *Trichoderma* sp. increases phenolic content more strongly but does not enrich flavonoids and triterpenoids simultaneously [63]; and *S. cerevisiae* fermentation improves physicochemical properties but has not been evaluated for digestion behavior [64]. *Wolfiporia cocos* therefore occupies a distinct position among SSF starters for wheat bran modification.

Furthermore, the mycotoxin and microbial indicators of the fermented product decreased after fermentation. However, because the transformation products of mycotoxins were not identified, and no direct toxicity assessment was performed in this study, the fate of mycotoxins during fermentation requires further investigation. In addition, the levels of heavy metals and pesticide residues were not determined.

This study has several limitations. The 17-day fermentation period is longer than that of conventional SSF starters; however, this extended duration is an intrinsic trade-off for the unique enhanced bioactive release achieved by *Wolfiporia cocos*. From a scalability perspective, the 17-day fermentation period is considerably longer than that typically required by conventional SSF starters such as *Aspergillus* spp. or lactic acid bacteria. This extended duration increases equipment occupancy, energy consumption, and the risk of contamination, and may limit throughput in industrial-scale production. However, the extended fermentation period was necessary to allow sufficient time for triterpenoid synthesis and extensive degradation of the bran cell wall, which are the key advantages of this fermentation system. Future work should explore strategies to shorten the fermentation period, such as optimizing the inoculum form (e.g., liquid seed culture), adjusting substrate composition, or adopting a two-stage fermentation strategy or using co-culture with a fast-growing starter in the early phase. Pretreatment of wheat bran (e.g., enzymatic or mild physical pretreatment) may also reduce the time required for cell-wall degradation. A full techno-economic assessment will be necessary before industrial application. The antioxidant capacity was evaluated solely by chemical assays (DPPH, FRAP, and reducing power), which have limited predictive value for biological effects such as bioavailability, cellular uptake, and in vivo activity. Individual bioactive compounds were not identified, and the apparent increases in total phenolics, flavonoids, and triterpenoids are based on colorimetric quantification. No cellular or in vivo validation was performed, and the digestion results were obtained using a static INFOGEST model. The correlations between bioactive compound release and antioxidant capacity were exploratory (*n* = 6, no correction for multiple comparisons), and causality cannot be established. In addition, the dry matter recovery after fermentation was not determined, and therefore a fermentation balance on a dry matter basis could not be established. As a result, the observed increases in the concentrations of crude protein, crude ash, water-soluble dietary fiber, and bioactive compounds cannot be definitively attributed to net synthesis or enzymatic release; part of these increases may result from the relative enrichment effect caused by substrate dry matter loss during fungal growth.

Future studies should address these limitations through compound-level identification by HPLC or LC-MS, Caco-2 cell assays and in vivo models, dry matter recovery measurements, sensory evaluation and stability studies, and optimization of the fermentation period through co-culture or pretreatment strategies.

In summary, *Wolfiporia cocos* solid-state fermentation modifies the chemical characteristics of wheat bran by increasing the concentrations of crude protein and soluble dietary fiber; showing a trend toward phytic acid reduction; enhancing the release of phenolics, flavonoids, and triterpenoids; and enhancing *in vitro* protein digestibility and bioactive compound release.

## Figures and Tables

**Table 1:** Experimental design and response values for response surface optimization.

Group	A: Inoculation Amount (%)	B: Solid–Liquid Ratio (*w*/*v*)	C: Fermentation Time (d)	Antioxidant Activity (μmol/g)
1	4	1:1.5	14	100.93
2	8	1:1.5	14	98.83
3	4	1:2.5	14	99.32
4	8	1:2.5	14	90.42
5	4	1:2.0	11	102.43
6	8	1:2.0	11	105.23
7	4	1:2.0	17	110.45
8	8	1:2.0	17	102.00
9	6	1:1.5	11	110.86
10	6	1:2.5	11	97.83
11	6	1:1.5	17	100.79
12	6	1:2.5	17	111.14
13	6	1:2.0	14	113.51
14	6	1:2.0	14	115.24
15	6	1:2.0	14	105.63
16	6	1:2.0	14	112.84
17	6	1:2.0	14	110.89

**Table 2:** Analysis of variance (ANOVA) for the fitted quadratic polynomial model of FRAP antioxidant activity in *Wolfiporia cocos*-fermented wheat bran.

Source	Sum of Squares	df	Mean Square	F Value	*p*-Value Prob > F	
Model	693.99	9	77.11	8.28	0.0054	significant
A—Inoculum Amount	34.65	1	34.65	3.72	0.0951	
B—Solid–Liquid Ratio	20.16	1	20.16	2.16	0.1847	
C—Fermentation Time	8.06	1	8.06	0.8655	0.3832	
AB	11.56	1	11.56	1.24	0.3020	
AC	31.64	1	31.64	3.40	0.1078	
BC	136.66	1	136.66	14.67	0.0065	
A^2^	217.50	1	217.50	23.35	0.0019	
B^2^	209.85	1	209.85	22.53	0.0021	
C^2^	1.48	1	1.48	0.1589	0.7021	
Residual	65.19	7	9.31			
Lack of Fit	10.61	3	3.54	0.2593	0.8518	not significant
Pure Error	54.58	4	13.64			
Cor Total	759.18	16				
R^2^	0.9141					
Radj^2^	0.8037					
CV%	2.90					
Adeq Precision	8.3808					

Note: A, inoculum amount (%); B, solid–liquid ratio (*w*/*v*); C, fermentation time (d). R^2^ = 0.9141; Adjusted R^2^ = 0.8037; Lack of fit = not significant (*p* = 0.8518).

**Table 3:** Changes in antioxidant activity of wheat bran induced by fermentation.

Methods		Group	
		CG	FWB
Antioxidant activity	DPPH radical scavenging rate (%)	37.00 ± 0.79 ^b^	58.96 ± 1.80 ^a^
	Total reducing power (μmol/g)	6.08 ± 0.17 ^c^	58.20 ± 1.77 ^b^
	Ferric reducing antioxidant power (μmol/g)	69.09 ± 1.59 ^d^	115.20 ± 3.12 ^a^

Note: CG, unfermented wheat bran (control group); FWB, fermented wheat bran. Data are presented as mean ± SD (*n* = 3 biological replicates). Different superscript letters within a row indicate significant differences (*p* < 0.05).

**Table 4:** Proximate composition of unfermented (CG) and *Wolfiporia cocos*-fermented (FWB) wheat bran.

Basic Composition (mg/g DW)	Group	
	CG	FWB
Crude protein	195.22 ± 2.78 ^d^	226.54 ± 3.36 ^c^
Crude ash	42.33 ± 1.16 ^b^	52.22 ± 1.51 ^a^
Crude fat	2.43 ± 0.05 ^a^	1.95 ± 0.06 ^b^
Moisture	6.98% ± 0.12 ^a^	6.72% ± 0.12 ^ab^
Cellulose	346.51 ± 5.78 ^a^	341.32 ± 11.55 ^a^
Soluble dietary fiber	43.20 ± 1.73 ^c^	87.53 ± 2.89 ^ab^

Note: CG, unfermented wheat bran (control group); FWB, fermented wheat bran. Data are presented as mean ± SD (*n* = 3 biological replicates). Different superscript letters within a row indicate significant differences (*p* < 0.05). All values are expressed on a dry-weight basis.

**Table 5:** Variation in phytic acid content in unfermented (CG) and *Wolfiporia cocos*-fermented (FWB) wheat bran.

	Indicator	Phytic Acid (μmol/g DW)
Group		
CG		8.72 ± 0.2 ^a^
FWB		7.15 ± 0.67 ^a^

Note: CG, unfermented wheat bran (control group); FWB, fermented wheat bran. Data are presented as mean ± SD (*n* = 3 biological replicates). The difference between CG and FWB was not statistically significant; the same superscript letter indicates no significant difference.

**Table 6:** Changes in contamination parameters of wheat bran prior to and following fermentation.

	Indicator	ZEN (µg/kg)	DON (µg/kg)	AFB1 (µg/kg)	OTA (µg/kg)	TBC (CFU/g)	TMC (CFU/g)
Group							
CG		<0.1	4.96 ± 0.12 ^a^	8.1 ± 0.19 ^a^	25.06 ± 0.59 ^a^	1.1 × 10^5^	3 × 10^4^
FWB		<0.1	2.2 ± 0.05 ^c^	7.33 ± 0.17 ^b^	21.93 ± 0.52 ^b^	2 × 10^4^	1 × 10^4^

Note: CG, unfermented wheat bran (control group); FWB, fermented wheat bran; ZEN, zearalenone; DON, deoxynivalenol; AFB1, aflatoxin B1; OTA, ochratoxin A; TBC, total bacterial count; TMC, total mold count. Data are presented as mean ± SD (*n* = 3 biological replicates). Different superscript letters within a row indicate significant differences (*p* < 0.05).

**Table 7:** Variation in antioxidant activity during simulated *in vitro* digestion.

Group	DPPH Radical Scavenging Rate (%)			Total Reducing Power (μmol/g)			Ferric Reducing Antioxidant Power (μmol/g)		
	Oral	Gastric	Intestinal	Oral	Gastric	Intestinal	Oral	Gastric	Intestinal
CG	40.70 ± 1.17 ^d^	48.84 ± 1.41 ^c^	54.13 ± 1.56 ^c^	3.11 ± 0.09 ^h^	9.33 ± 0.22 ^g^	15.55 ± 0.45 ^f^	61.49 ± 1.77 ^g^	64.94 ± 1.87 ^fg^	98.11 ± 2.83 ^e^
FWB	61.44 ± 1.77 ^b^	73.72 ± 2.13 ^a^	76.79 ± 2.22 ^a^	30.32 ± 0.87 ^e^	36.38 ± 01.05 ^d^	60.64 ± 1.77 ^a^	103.20 ± 2.97 ^cde^	108.99 ± 3.11 ^cd^	164.65 ± 4.75 ^a^

Note: CG, unfermented wheat bran (control group); FWB, fermented wheat bran. Data are presented as mean ± SD (*n* = 3 biological replicates). Different superscript letters within a row indicate significant differences (*p* < 0.05).

**Table 8:** Pearson correlation coefficients between bioactive compound release and antioxidant capacity during *in vitro* digestion.

Indicator	DPPH Radical Scavenging Rate	Total Reducing Power	Ferric Reducing Antioxidant Power
Total phenolic release	r = 0.974 **; r^2^ = 0.949	r = 0.959 **; r^2^ = 0.920	r = 0.967 **; r^2^ = 0.935
Flavonoid release	r = 0.850 *; r^2^ = 0.723	r = 0.973 **; r^2^ = 0.947	r = 0.969 **; r^2^ = 0.939
Triterpenoid release	r = 0.624; r^2^ = 0.389	r = 0.846 *; r^2^ = 0.716	r = 0.712; r^2^ = 0.507

Note: * indicates significant correlation at the 0.05 level (two-tailed); ** indicates significant correlation at the 0.01 level (two-tailed); *n* = 6. r^2^ represents the coefficient of determination. These correlations are exploratory and hypothesis-generating; no correction for multiple comparisons was applied, and causality should not be inferred.

## Data Availability

The original contributions presented in this study are included in the article. Further inquiries can be directed to the corresponding author.
